# Patient involvement in the development of patient-reported outcome measures: The developers’ perspective

**DOI:** 10.1186/s12913-017-2582-8

**Published:** 2017-09-08

**Authors:** Bianca Wiering, Dolf de Boer, Diana Delnoij

**Affiliations:** 1 Tranzo (Scientific Centre for Transformation in Care and Welfare), Tilburg University, PO Box 90153, 5000 LE Tilburg, The Netherlands; 2 NIVEL (Netherlands institute for health services research), Utrecht, the Netherlands

**Keywords:** Patient-reported outcome measures, Patient participation, Questionnaire development procedures, Qualitative research

## Abstract

**Background:**

Patient-reported outcome measures (PROMs) are increasingly used in health care. To capture the patient’s perspective, patient involvement in PROM development is needed. As earlier research showed varying degrees of patient involvement in PROM development, this study aimed to investigate why PROM developers do or do not involve patients, how patients can be successfully involved and what the negative aspects and benefits of patient involvement are.

**Methods:**

PROM developers who, according to an earlier scoping review, involved patients in at least two phases of PROM development or did not involve patients at all, were contacted for a telephone interview. The interviews were recorded, transcribed and analysed using a general inductive approach.

**Results:**

From the PROM developers who involved patients, 21 developers were interviewed and three answered questions via e-mail. Most developers considered patient involvement necessary to create a valid questionnaire and relied on guidelines, personal experience and practical considerations for choosing a qualitative method. Negative aspects of patient involvement were mainly time investment and budget impact. One developer who did not involve patients was interviewed. Two developers sent back answers via e-mail. These developers did not involve patients because of limited resources or because no benefits were expected.

**Conclusion:**

Although PROM developers agree that patient involvement is necessary, a lack of resources can be a stumbling block. Most developers rely on guidelines, personal experience or practical considerations for choosing a qualitative method. Although this may be a good place to start, to optimize patient involvement developers should explicitly think about which methods would suit their study.

## Background

Patient-reported outcome measures (PROMs) are questionnaires which measure patients’ perspectives on health outcomes [1, 2]. PROMs may be used to inform patients of the performance of treatments and health care professionals, improve the care of individual patients, assist health care purchasers and reward performance in pay for performance schemes [2–5]. PROMs are increasingly assigned these important tasks of measuring performance, treatment progress and quality of care in health care systems such as the English National Health Service (NHS) [2, 4], the American Centers for Medicare and Medicaid Services (CMS) [6], the Dutch and the Swedish health care system [7].

As PROMs are meant to reflect the patient’s perspective, the aim should be to use valid PROMs which truly reflect this perspective. Patient involvement throughout the development of PROMs is therefore essential [8–13]. Patient involvement can have a great impact on the questionnaire as the relevance of outcomes and the comprehensibility of the questionnaire can only be determined by patients [12, 14–19]. Furthermore, patients may fail to complete the questionnaire if the questionnaire does not sufficiently reflect the patient’s perspective. A lack of patient involvement may have a negative influence on the validity, sensitivity and response of a questionnaire [13, 20].

However, although patient involvement in PROM development has been recommended by researchers [8–12, 21], and has also become a requirement of the US Food and Drug Administration (FDA) [22], many PROMs appear not to have been developed with patient input [9, 23, 24]. A scoping review of 193 PROMs looking at whether and to what extent patients are involved in the development of PROMs, suggests that in over a quarter of the included papers describing the development of a PROM, no patient involvement was recorded. Furthermore, the level of patient involvement in the remaining PROMs varied greatly, from feedback forms to focus groups, interviews and cognitive interviews [24]. Another review of 42 PROMs for rheumatoid arthritis showed that PROMs only minimally covered personal factors which determine functioning and the individuals’ experience of disability according to the International Classification of Functioning, Disability and Health (ICF) and which were deemed important by patients [25]. Other studies which investigated patient involvement in a small number of PROMs suggest a complete lack of patient involvement [23], conceptual difficulties and questionnaire design problems. These problems might have been prevented by involving patients in the development process [9].

Apparently, not all PROM developers choose to involve patients in the development of their PROM. Besides, if a developer does decide to involve patients, there is a great variety in the levels and methods of involving patients. Although many benefits may be gained by involving patients [26], research suggests that developers also need to consider slightly less positive consequences such as the costs associated with patient involvement [12, 26]. Additionally, there is no perfect method to involve patients, as fairly little is known about which methods are the most successful and which circumstances are needed for achieving optimal outcomes [27]. Decisions that need to be made, such as whether patients should be involved, to what extent patients should be involved, and which methods should be used to involve patients, appear to be mostly based on the experiences and opinions of the researchers, the organisation they belong to, or the regulations or guidelines they adhere to such as the US Food and Drug Administration [22] or PROMIS (Patient-Reported Outcomes Measurement Information System**)** [28].

However, as most professionals involved in PROM development deal with similar decisions, it is necessary that more insight is gained in how patients can be successfully involved in PROM development and what kind of benefits and costs of patient involvement are experienced by PROM developers. By looking at successful involvement, we are not necessarily interested in how patients were involved, as this was also recorded in an earlier scoping review [24]. The focus lies instead on how developers chose their methods, and how they experienced using these methods. Such an overview may help PROM developers make a more informed decision regarding patient involvement. This qualitative study therefore aimed to gather the views of PROM developers, who did and did not involve patients, regarding patient involvement and its negative aspects and benefits. Our research questions are:

According to the interviewed PROM developers:

1. Why did certain developers involve patients and others did not?
2. How are patients successfully involved in the development of PROMs?
3. What do patients contribute to the development of PROMs?
4. What are negative aspects of patient involvement?

## Methods

### Participants

PROM developer recruitment was based on the list of PROMs made for an earlier review [24]. The list was composed by systematically searching the databases PubMed, Embase, MEDLINE and the Cochrane Methodology Register for papers describing new PROM developments. Patient involvement in three phases of PROM development was recorded. For the present study PROM developers were contacted who, according to the review, involved patients in at least two phases of development or did not involve patients at all. Patient involvement during at least two development phases was chosen because involving patients during two or more development phases means that developers have extensively involved patients, usually using several methods. Developers are therefore able to discuss several patient involvement experiences and compare methods in terms of usefulness and experienced problems. Developers who involved patients during less than two development phases were not included, as we did not think they would provide additional and unique information in addition to the experience of developers who involved patients in multiple phases of development. Recruitment was limited to the PROM study publication dates between 2008 and May 2014 to ensure good remembrance of the development process. The author named as contact in the publication included in the review was contacted.

### Procedure

A list of questions was drafted to guide the interview process (Tables 1 and 2). The questions were tested in three pilot interviews with researchers who had experience with patient involvement during questionnaire development. No changes were made to the list of questions.

**Table 1:** Questions used as interview guide for interviews with developers who involved patients

Questions for developers who involved patients
*Patient recruitment*
-Why did you decide to involve patients in the development of your questionnaire?
-How many patients were involved in your development process?
-How did you decide how many patients you wanted to involve?
-How did you recruit the patients who were involved in your study?
-Did you have any difficulties with the recruitment of patients?
-Would you consider the patients who were involved a good representation of the patient group?
*Methods of involving patients*
-How many methods did you use to involve patients in the development of your questionnaire?
-During which development phases did you involve patients?
-Which methods did you use to involve patients?
-How did you decide which methods to use?
-The participation ladder of decision making in health care consists of three steps. At the first step patients are involved as consultants. At the second step patients are considered partners and at the third step patients have a dominant role. Which step of the ladder describes the position of the patients in your development process the most accurate?
-Did you have any difficulties with involving patients in your development process?
-If focus groups were used, how many focus groups were organised and how many patients were involved per focus group?
-If interviews were used, how many interviews took place?
-Was a specific method used to interview the patients?
-If cognitive interviews were used, how many interviews took place?
-If another method was used; how many patients were involved?
-Which method was the most successful and why?
-Which method was the least successful and why?
*Benefits and negative aspects of patient involvement*
-Did patient involvement influence the questionnaire in any way?
-If you could go through the development process again, would you change anything concerning patient involvement?
-Were there any benefits from involving patients? What kind of benefits?
-Did you perceive any negative aspects concerning patient involvement? What kind of negative aspects?
-In hindsight, was there anything that could have been done to prevent these negative aspects?
-Did the benefits outweigh the costs?
-Would you recommend involving patients during the development process?

**Table 2:** Questions used as interview guide for interviews with developers who did not involve patients

Questions for developers who did not involve patients
-Was involving patients in the development of the questionnaire actively considered?
-Was there anything you expected to gain from patient involvement?
-Where there any negative consequences you expected to experience regarding patient involvement?
-What were the main reasons for not involving patients?
-Would you consider the questionnaire relevant for patients?
-Could the questionnaire have been improved by involving patients?
-If you would develop a new questionnaire, would you involve patients?
-How did you make sure that patients interpreted the questions as they were intended?
-How did you make sure that patients could complete the questionnaire?

PROM developers were sent an e-mail asking whether we could contact them via telephone for some questions regarding patient involvement during the development of a certain questionnaire. Reminders were sent a few weeks later. If PROM developers agreed to a telephone interview, a questionnaire (Tables 1 and 2) was sent and an appointment was scheduled. If PROM developers answered the questions via e-mail, an e-mail was sent informing the developers of the purpose of our study. In this e-mail we also asked them to consent to the use of the information for a paper. PROM developers who agreed to a telephone appointment were phoned at the scheduled time and date and were informed of the purpose of our study. They were asked to consent to the recording of the interview and the use of the information given during the interview for a paper. The conversation and informed consent were recorded. A semi-structured approach [29] was used for the interviews. Although the questions were used to guide the conversation, room was created for other topics. Transcripts of the interviews were sent to the PROM developers for approval. 17 of the 22 developers returned their transcript with no or small textual alterations. One developer added some text further explaining his/her methods.

### Analysis

The interviews were transcribed verbatim, after which the transcriptions were analysed using a general inductive approach for analysis of qualitative evaluation data [30]. Segments of the interview text were coded closely following the meaning of the segments, after which the segments were grouped into subthemes and themes. For example, when asked for the difficulties of patient involvement, a developer explained that patient involvement is costly and takes time. The subthemes would be ‘costs’ and ‘time’. All subthemes describing difficulties or challenges would be grouped under the theme ‘challenges of patient involvement’. The transcripts were analysed using ATLAS.ti, version 7 [31]. All interviews were analysed by author BW. The first three interviews were checked by author DD to ensure that no relevant information was missed and the coding matched her understanding of the content. Five interviews were double coded by authors BW and DB to check the reliability and transparency of the coding. Any differences were discussed until agreement was reached. Finally, a content check was performed where authors DD and DB each read 10 interviews and made sure that the interviews were well reflected in the results section.

## Results

### Response

Forty-one PROM developers who involved patients and 16 PROM developers who did not involve patients were invited to participate. Initially 22 PROM developers who involved patients agreed to an interview. Seventeen developers who involved patients did not respond to the invitation. One interview took place at the developer’s place of work, the other interviews were held via telephone. One interview was joined by another developer involved with the instrument. This resulted in 21 interviews. Additionally, three PROM developers who involved patients sent back the completed questionnaire. This is a response rate of 58.5%. Of the 16 PROM developers who did not involve patients only two developers sent back a completed questionnaire and two developers agreed to an interview. Twelve developers did not respond to the invitation. After the interviews had taken place one PROM developer withdrew his/her consent. This is a response rate of 18.8%. Most participants were native English speakers from the United Kingdom, the United States, Canada and New Zealand. One participant came from Germany. Most non-respondents also came from the United States or the United Kingdom. However, as ten out of eleven non-native English speakers declined our invitation, there was a difference in countries of residence between respondents and non-respondents. The average publication date of respondents was 2011, which was similar to the average publication date of non-respondents.

#### Themes

The major themes that were identified during the analysis of the interviews concerned the motivation for involving patients, motivation for not involving patients, requirements for patient involvement, the benefits of patient involvement, the overall challenges of involving patients, recruitment methods, recruitment challenges, methods to involve patients, method motivation, challenges with methods, and future plans for patient involvement. Although many hours of interview recordings were collected, in this paper only the interview sections are discussed which are considered highly relevant and essential to answer our research questions. The selection of interview sections was based on the themes which were derived from the interviews.

#### Why did developers involve patients?

##### Motivation for involving patients

There are many reasons why developers felt that they had to include patients. Some developers involved patients because this was required by the FDA. Many developers wanted to understand how it feels to live with a certain disease or condition. Furthermore, developers wanted to be sure that their PROM covered all the important issues, was relevant to patients and could easily be completed by patients. One developers said that it is the academic’s role to voice what patients want and experience.

“*I don't know what it is to live with rheumatoid arthritis. They have fatigue caused by rheumatoid arthritis, so I could not do it better on my own. I would say that we are the voice of the patient. The patients know what they want and we as academics have to be their voice and develop it. But it has to come from them.”*

One researcher gave an example illustrating why it is important that PROMs are relevant to patients:

*“It is called a generic measure* (EQ-5D*), but of course the five aspects of health that are selected are more relevant to some conditions than they are to others. So someone with macular disease, whose sight is severely impaired and registered as what used to be called blind may appear to be in perfect health on the EQ-5D, because it doesn't affect the five aspects of health that are being asked about. Yet their quality of life is shot to pieces, because they can't see. And yet, NICE is demanding EQ-5D data from pharmaceutical companies evaluating new treatments*.*”*

##### Future plans for patient involvement

Regardless of how beneficial patient involvement was or how many difficulties related to patient involvement PROM developers experienced, all PROM developers would recommend patient involvement and wished to involve patients again during their next PROM development.

*“… it is like saying would you do research without a statistician.”*

#### How are patients successfully involved in the development of PROMs?

##### Requirements for patient involvement

Successful patient involvement requires trained and/or experienced staff, good communication, a clear understanding of roles and expectations and awareness of a patient’s limitations.

*“I think the key thing once again is being clear from the outset about roles and expectations around how things work. We were very clear about that.”*

##### Recruitment methods

Most developers recruited patients in clinics or other health care centres. Other more common options were charities and recruitment companies. Most developers recruited until data saturation was reached.

##### Recruitment challenges

The biggest challenge of recruiting patients was to achieve diversity, as finding patients with certain characteristics could be difficult. Examples of these characteristics were different cultures, age groups or patients with disabilities. However, most developers were confident that they had achieved a good representation of the patient population.

*“Well obviously by the end you knew you need to find a 25 year old man and that is quite difficult in arthritis. It is mostly middle-aged women and by the end of it you are always looking for a young man at the end.”*

Another difficulty was getting patients to actually participate. As patients are managing a personal life, work life and an often time and energy consuming disease, patients are not always able to or interested in attending interviews or focus groups. Developers therefore tried to encourage participation by fitting appointments between hospital appointments, offering lunch and keeping patients updated. One developer mentioned difficulties with people lying about their condition as they then got paid for participating. Another developer encountered problems when he/she wanted to investigate an issue that is taboo among a certain culture. Even if patients were willing to participate, family members sometimes kept them from participating.

##### Methods used to involve patients

Most developers involved patients during item development and while testing the PROM for comprehensibility. The most used methods to do this were interviews and/or focus groups. Sometimes focus groups were organised online. Slightly less common but still much used methods were cognitive interviews and feedback questionnaires. Some developers involved patients as patient research partners. When asked to choose their preferred method, most developers said that this is impossible as the methods serve different purposes and complement each other.

##### Method motivation

Many researchers used scientific guidelines or standards as a guide for patient involvement.

“*I mean, our whole development process has been informed by FDA guidelines, so that is how we decided to use the methods we have used. The FDA guidelines are exactly that. They are guidelines. They are not strict rules so they are open to certain amount of interpretation. But certainly our development process complies with their guidelines.”*

Literature, personal preferences and experiences and practical considerations such as how much time or money it costs and how difficult it is to organise also influenced the method of patient involvement. Focus groups were sometimes chosen because they were an easier and cheaper way to receive the input from many patients at once.

*“The reason why we choose focus groups for […] was in part driven by cost implications, because it makes it easier to include a larger number of patients for less cost.”*

Additionally, the interaction between patients was greatly appreciated as developers thought that it would result in different or more information than interviews. Interviews, however, were chosen because they offered a one on one opportunity with the patient. This not only made it more suitable for private subjects, but it also resulted in richer data.

“*We’ve always done individual interviews because we find we get a lot better, or more information, rich information*.”

Furthermore, developers considered interviewing an easier method to involve patients.

##### Challenges with methods

Although developers reported few difficulties with interviewing, a developer did mention that it can be challenging if a patient is not very reflective or talkative and that patients can get off-track. However, developers experienced more difficulties with focus groups. Focus groups mainly suffered under dominant patients taking over the focus group and patients getting off-track. These problems were solved by removing a patient, intervene verbally or use experienced moderators.

“*We would actually move that patient out of the focus group, if we couldn’t stop them any other way. Sometimes these are personality traits, and it’s not that we’re there to blame that person, so we might then have to help to say this, very difficult patient, mister X, you’ve got such comprehensive views on this, I think it would be very helpful if you came with me and sat with me in a separate room and tell me all about this*.”

#### What do patients contribute to the development of PROMs?

Patients mainly contributed to the questionnaire development by helping to increase the validity and comprehensibility of the PROM. Patients were able to offer a different perspective than physicians.

“*In diabetes care until five years ago if you asked any clinician or diabetologist: “How important are non-severe hypoglycaemic events?” They would say: “Not important at all. Drink a glass of orange juice. You are fine.” We don’t even think about it. But by talking to patients we found out that non-severe events had a huge impact on patients’ functioning and well-being. Because after they drink the orange juice they still feel terrible for 20 h*.”

Involving patients ensured that the PROM was relevant to patients, and that patients were more likely to complete the PROM.

*“We hadn't asked any questions about changes in medication in the PROM before and after treatment. Some patients put notes on their responses saying: “I have been able to stop taking my medication”, which was something we hadn't considered.”*

Many developers also highlighted that it is a great experience for patients as patients can share experiences with each other, learn from each other, and also feel valued by being involved.

*“Yeah, you know, I think especially for the focus groups. We found that people tended to have a very positive experience. We definitely had people who even, after the groups would exchange contact information with each other.”*

#### What are negative aspects of patient involvement?

On the downside involving patients is time consuming, logistically challenging and impacts the budget. It can also add to the complexity of the research project and can be difficult to report. Furthermore, involving patients may be upsetting for both patient and developer.

*“So again from the practical viewpoint it is financially and time consuming. And as I said it was upsetting in some cases, but I think overall we felt that patients were getting a lot out of it.”*

Some developers also mentioned that patients were only able to reflect their own, personal perspective, which could influence the relevance of the research if only a few patients were involved and/or if a patient was involved extensively. These challenges however, would not deter most developers from involving at least a few patients.

#### Why did some developers choose not to involve patients?

The PROM developers who refrained from involving patients mainly did so because they either did not have the time and resources or did not expect to gain anything. Even though their aim was to develop a PROM, one developer said that they did not aim to capture the patients’ perspective. Another developer considered patient involvement unnecessary as the disease was well documented. Additionally, he/she felt, based on earlier experiences, that patients mostly just added subjective impressions of single patients.

*“From earlier questionnaire developmental processes we got the impression that patient involvement introduced several additional aspects. However, many of those aspects were very subjective impressions of single patients without significant impact on the questionnaire.”*

##### Plans for patient involvement in future developments

Even though not all developers were convinced of the need to involve patients during PROM development, all developers thought that patient involvement could be beneficial, dependent upon circumstances such as the aim of the questionnaire and the availability of resources. Therefore they would consider involving patients next time.

## Discussion

As the importance of patients’ knowledge on health and health care as a source for improving the quality of care is increasingly recognised [2, 4, 11], PROMs are used more and more [1, 2, 4]. Although for capturing patients’ perspectives on health care patient involvement in PROM development is deemed necessary [8–12], patients are still not always involved [24]. There is not one correct procedure for involving patients [27] and choosing between patient involvement and no patient involvement can be difficult as both can have consequences for the research project. This study therefore aimed to give more insight into PROM developers’ experiences with patient involvement by first investigating how patients are involved in the development process. Patients were mostly recruited via clinics or health professionals until data saturation was reached. Recruiting until data saturation has been reached is a method in qualitative research that is widely accepted and used by many researchers [32, 33]. The developers mostly involved patients using interviews and focus groups. These methods are mentioned by several scientific standards or guidelines as fitting methods to involve patients [28, 34]. Although some developers indicated that they adapted the recommended methods to their patient group or to how they thought it would work better, many followed the guidelines or used methods which were the most practical and/or cheapest, or based their choice on personal experiences and preferences.

Besides the more common methods used to involve patients, several developers involved patients especially in their more recent PROM developments as patient research partners or included patients in steering or advisory groups. Even though this more collaborative form of patient involvement may have some issues that need to be dealt with, such as the amount of experience and skills patients would need to actively participate [35] and whether patients who are able to participate to such an extent are good representatives of the patient group [36], it is considered an important step forward for patient involvement in PROM development [12].

Another less common but increasingly used method concerns conducting qualitative research on the internet. As many people use the internet, conducting qualitative research online can be helpful in gathering the opinions of many patients [37]. Patients with rare conditions can also be more easily reached [38] and communicating online may offer patients more freedom to express themselves [39]. Online qualitative research also has some drawbacks. For example, you are only able to study a select group of patients as patients need to have access to internet and certain skills to participate on the internet [40, 41]. Additionally, it is much less clear what the rules are regarding informed consent and what is considered a private conversation and what is not [37]. As both online and face to face qualitative methods have several pros and cons, a decision for either of these methods should be made separately for each study.

Besides how patients are involved in PROM development, the present study also investigated what patient involvement contributes towards the PROM development. Patients contributed by offering their experiences and views regarding living with a disease. These views not only helped increase the validity and comprehensibility of the PROM, but also helped to ensure that the PROM was relevant for other patients. The benefits of patient involvement were very important to PROM developers as most would not consider to refrain from involving patients, even if they did not really have the budget or the time to do it.

Although most PROM developers were overwhelmingly enthusiastic about patient involvement, negative aspects and costs of patient involvement were encountered at several levels. Most developers experienced some difficulties finding enough diverse patients and getting patients to fit research into their lives could be challenging. Furthermore, actually involving patients could be challenging as most developers encountered patients who dominated the conversation or easily went off-track. Overall, patient involvement was time consuming, made an impact on the budget and was logistically challenging. Although the costs of patient involvement are sometimes recognised in the literature as a challenge of qualitative research [12], the challenge of actually recruiting and involving patients and the time it takes to do it well should also be acknowledged.

It should therefore come as no surprise that a lack of resources was one of the reasons why not all PROM developers involved patients. There were however also a few PROM developers who did not see any added benefit of patient involvement for their PROM. As patient involvement is increasingly required by organisations such as the FDA [34], these PROM developers may not have this choice for much longer.

### Limitations and strengths

Some limitations need to be taken into account. First, this study aimed to give insight into the views of both developers who involved patients and developers who did not involve patients. However, only a low number of PROM developers who did not involve patients were willing to answer our questions.

Second, only 58.5% of the invited PROM developers who did involve patients agreed to participate in our study. As we indicated in our invitation that we wished to discuss patient involvement in PROM development, it is possible that the PROM developers who agreed to participate were perhaps more enthusiastic about patient involvement than the PROM developers who did not reply. Whether we received a reply was in any case not related to how long ago the paper was published, as no difference was found between the publication dates of developers who participated and developers who did not participate. However, developers may have felt unsure about discussing their research in a language other than their native language, as all but one non-native English speakers declined our invitation. This makes it difficult to generalise the results beyond English speaking countries.

Third, results may be influenced by a confirmation bias. As patient involvement costs time, resources and effort, developers must have been very motivated to involve patients. This may also mean that they are more focused on the positives.

Fourth, although by including both co-authors in the analysing process efforts were made to guard the transparency and reliability, results may be coloured by views held by the first author who interviewed the developers and was the main coder.

An important strength of this study is that we were able to offer an insight into the views of many both experienced and less experienced PROM developers who worked with a number of patient groups and developed a PROM fairly recently. This study was therefore able to not only give insight into the methods used to recruit and involve patients, the many pitfalls and benefits of patient involvement, but also into the general opinion held by PROM developers regarding patient involvement in PROM development.

### Implications

The results of this study have several implications for patient involvement in PROM development. First, this study indicates that patient involvement requires the availability of resources such as time and money. A lack of or limited resources may keep developers from optimally involving patients or it may keep them from involving patients at all. As patient involvement is included in some scientific guidelines [28] and the Food and Drug Administration even requires it [34], it is essential that the necessary resources are included in the costs and the duration of the study when applying for subsidies so this can no longer be a stumbling block.

This is especially important as not involving patients may have implications for the use of the PROM. It may impact the response and the validity of the PROM [13, 20]. Perhaps more importantly, PROMs are used for important purposes, such as measuring the performance of health care providers [2–5]. Not involving patients may mean that health care providers are judged on aspects of care which may not be important to patients, while important aspects may be forgotten. Furthermore, research shows that health care providers especially try to improve on the performance aspects they scored less well on [42]. If performance measures are used which do not reflect what patients consider important, health care providers may neglect to improve on aspects of care which are important to patients. Because of the possible consequences of using PROMs which do not reflect the patient’s perspective adequately, patient involvement should be taken very seriously.

Furthermore, the results suggest that many developers keep to scientific guidelines for methodological decisions or alternatively pick methods to involve patients which are most practical to them or which suit their personal preferences. Guidelines may be a good place to start as it guarantees a certain level of patient involvement and practical solutions should certainly not be completely disregarded as it helps developers involve patients within their limitations. However, as there is no perfect method for involving patients [27], it may be more beneficial that pros and cons of the available methods are considered. For example, based on our results, interviews may be especially fitting for discussing intimate or taboo subjects. Focus groups may be helpful to learn about the majority view or if you have a very tight budget. Both also have drawbacks such as the difficulty of keeping a focus group on track, or the single subjective view of a patient during interviews. It is therefore important that a method is picked based on what is most suitable for the patient group and the purpose of the study.

Fourth, several PROM developers mentioned the importance of good qualitatively trained and/or experienced staff for moderating focus groups or conducting interviews. They also highlighted the importance of good communication, a clear understanding of roles and expectations and awareness of patient’s limitations. These requirements combined with the difficulties one may experience while interviewing or conducting focus groups asks a lot of the persons who are hired to do so. Adequately training staff to deal with patients and conduct qualitative research may be imperative for successful patient involvement and optimal patient benefits for the PROM development.

## Conclusion

Even though patient involvement may be difficult at times as it is costly, time consuming, logistically challenging and some patients can be hard to handle, all PROM developers who involved patients during PROM development agree that patient involvement in PROM development is necessary. Patient involvement results in a more valid and comprehensible PROM which is more relevant to patients. Although most developers are united in their enthusiasm for patient involvement, a lack of resources can be a stumbling block. Additionally, this enthusiasm for patient involvement does not appear to stretch out towards choosing the methods for involving patients. Most developers follow guidelines or standards or choose a method based on personal experience or practicalities. They do not always appear to actively consider pros and cons of different methods that can be used to involve patients. Although guidelines for PROM development, personal experience and practicalities may be a good place to start, to optimize patient involvement developers should explicitly think about which methods would suit their study and the patient population of interest.

## Abbreviations

CMS: Centers for Medicare and Medicaid Services
EQ-5D: EuroQol five dimension scale
FDA: Food and Drug Administration
ICF: International Classification of Functioning, Disability and Health
NHS: National Health Service
NICE: The National Institute for Health and Care Excellence
PROM: Patient-reported outcome measure
PROMIS: Patient-Reported Outcomes Measurement Information System
